# Case of Hydrops Articuli

**Published:** 1862-06

**Authors:** S. Barrett

**Affiliations:** Le Roy, N. Y.


ART. III.—*Case of Hydrops Articuli, by* S. BARRETT, M. D., *Le Roy, N. Y.*

U. A., aged 21, came into my Infirmary April 21, 1861, with the cavity of the right knee joint distended with fluid so as to separate the patella from the bones of the joint; the swelling extended considerably above and below the joint. The history of the case was, that several years since he met with a severe sprain of the joint, which was followed by great soreness and inflammation, which laid him up for several weeks; when the soreness was so far removed that he was enabled to move about with crutches, he began to discover slight fluctuation through the joint, and enlargement; as the soreness passed away, about two years since, the enlargement remained and gradually increased; at times he would be obliged to suspend all exercise. When he presented himself, fluctuation was very distinct through the joint; the capsule and ligaments were considerably thickened, which gave feeling and appearance, as though the bones were enlarged. His constitution and general health not being seriously impaired, I decided to puncture the joint, draw off the fluid, and if too high degree of inflammation did not follow in a few days, inject with solution of iodine. I accordingly made a valvular incision a little upon the inside of the patella, which was followed by a sudden gush of about four ounces of gelatinous fluid; the limb was placed in an elevated position, and kept at rest, with a bandage applied as firmly as the joint would bear. Three days after, with a hypodermic syringe, I punctured the joint upon the inside of the knee, and injected ⅛ ʒ of solution of iodine, of the following strength: Iodine ʒi; Iodide potass ʒi; Aqua pura ℥i. Considerable inflammation followed; the joint swelled pretty full; in a few hours became hot and painful; the limb was kept elevated; evaporating lotions were applied, so as to keep down inflammation, and a bandage applied as soon as it could be borne; in six weeks there was found to be some increase of fluid in the joint. The operation was repeated in the same manner as the first, injecting the iodine into the outside of the joint; this arrested the morbid secretions. He was discharged the first of July, the knee about the size of the other, free from any soreness or unnatural effusion; the joint has been sustained by a knee-cap, and gradually recovering strength; at this time it is as limber as the other, and he has resumed his labor upon a farm.

